# Blockade of Dopamine D3 Receptors in the Ventral Tegmental Area Mitigates Fear Memory Generalization

**DOI:** 10.3390/ijms26136520

**Published:** 2025-07-07

**Authors:** Xiangjun Fang, Xiaoyan Ding, Ning Wu, Jin Li, Rui Song

**Affiliations:** 1 Nanjing University of Chinese Medicine, Nanjing 210029, China; fangxj1025@163.com; 2 Academy Military Medical Sciences, 27th Taiping Road, Beijing 100850, China; elaine972@163.com (X.D.); jinli9802@163.com (J.L.)

**Keywords:** fear generalization, ventral tegmental area, dopamine D3 receptor, basolateral amygdala

## Abstract

The generalization of fear memories is an adaptive neurobiological process that promotes survival in complex and dynamic environments. While generalization has adaptive value, fear generalization is maladaptive and is a significant feature of stress-related disorders such as post-traumatic stress disorder (PTSD). The dopamine system plays a crucial role in both reward- and fear-related processes; however, the contribution of dopamine D3 receptors (D3Rs) to fear generalization in intense foot-shock models remains unclear. In this study, we administered a highly selective D3R antagonist, YQA14 (1 μg/0.2 μL/side), in the ventral tegmental area (VTA), which significantly inhibited fear generalization in novel contexts within foot-shock models. This effect was mediated by reducing the neuronal activity in the basolateral amygdala (BLA). These findings enhance our understanding of the neurobiology of generalization, which is essential from a translational perspective and has broad implications for treating generalized fear disorders.

## 1. Introduction

Fear generalization is defined as the loss of the ability to accurately associate threat signals during fear learning, resulting in excessive defensive reactions [1]. Research indicates that excessive fear generalization, characterized by exaggerated fear responses to safe stimuli, is a significant pathological marker of post-traumatic stress disorder (PTSD) [2]. Beyond PTSD, other emotional disorders, such as social anxiety disorder, also exhibit fear generalization; for example, patients may feel anxious in numerous social situations due to a single negative experience [3]. Through research, it is possible to identify potential therapeutic targets to address maladaptive fear responses by elucidating the biological underpinnings of fear generalization.

Current research has highlighted the neural circuitry of fear generalization, which involves the mesolimbic dopamine system, including the hippocampus, amygdala, insula, and medial prefrontal cortex [4]. The amygdala is central in fear learning and generalization [5,6]. For instance, Ciocchi et al. demonstrated that activating neurons in the lateral part of the central amygdala or inhibiting neurons in the medial part of the central amygdala enhances the fear response to a conditioned stimulus [6]. Similarly, Resnik and Paz reported that neuronal changes in the basolateral complex of the amygdala (BLA) impair discrimination learning during the generalization phase in primates [7]. Critically, the dopaminergic projection from the ventral tegmental area (VTA) to the BLA has emerged as a key modulator of fear-related behaviors [8,9]. Recent studies have shown that inhibiting dopaminergic projection inputs to the BLA impairs fear acquisition, whereas the chemogenetic activation of this pathway enhances fear responses [9]. While these studies have delineated the structural and functional roles of the projection from the VTA to the BLA, the molecular mechanisms through which dopamine (DA) modulates fear generalization require further elucidation [10].

Not only do the ventral midbrain dopamine neurons provide signals related to the value and salience of stimuli during Pavlovian associative processes, they also play a key role in fear discriminative learning [11]. As the main neurotransmitter of motivational control, dopamine can evaluate the beneficial or aversive nature of stimuli and guide individuals in choosing their approach or avoidance behavioral responses. Studies have shown that stress and aversive experiences lead to significant changes in dopamine concentrations, which affect an individual’s behavioral responses to aversive stimuli [12,13]. Research has revealed diversity in the activity patterns and functions of DA neurons. DA neurons can be divided into multiple populations, each encoding different motivational signals. One population encodes the value of stimuli, while another is associated with stimulus salience. For responses to aversive stimuli, the activity of DA neurons shows different patterns of excitation or inhibition among different groups [14]. These findings challenge the traditional view that DA neurons uniformly transmit motivational signals.

Dopamine is critically involved in fear memory, including the formation, storage, and extinction of fear memories via binding to specific dopamine receptors [15]. In stress-related disorders, such as PTSD, dopamine system dysfunction manifests as altered dopamine levels and receptor dysregulation [11,16]. Preclinical studies further support this link; Zweifel et al. showed that NMDA receptor inactivation weakens dopamine-dependent fear extinction [17]. Notably, the VTA→BLA dopaminergic pathway is pivotal in fear memory formation and anxiety generalization. Dopamine, the primary neurotransmitter in the VTA, modulates anxiety through dopamine D1 and D2 receptors, suggesting that these receptors are involved in fear generalization mechanisms [2]. Emerging work in psychiatric disorders is characterized by dysregulated fear responses [18]. Zweifel [2] showed that the inhibition of dopaminergic neurons impaired the establishment and expression of discriminative fear memories, concurrently triggering persistent fear generalization and anxiety. The optogenetic activation of dopamine neurons was further shown to effectively reduce fear generalization. In addition, Corral-Frias [19] observed that, in a rat model of PTSD, intense stimuli induce sustained VTA dopaminergic neuron hypoactivity, driving persistent maladaptive behaviors.

The functions of dopamine in the brain are complex and diverse, primarily exerting their physiological effects through different subtypes of G protein-coupled receptors. Different dopamine receptor subtypes exert significantly different effects on their functions. D1-like (D1 and D5) receptors bind to the G protein Gs and activate adenylyl cyclase, while D2-like (D2, D3, and D4) receptors have the effect of inhibiting adenylyl cyclase [15,16]. In the mesolimbic DA system, the expression density of D3 receptors (D3Rs) is low, but their importance in controlling emotional responses, reward motivation, and executive function is, over time, increasingly recognized. D3Rs are considered to be associated with various mental health disorders (such as schizophrenia and depression) and play an important role in regulating fear-related associative learning and anxiety-like behaviors [17]. Their distribution pattern suggests that they could represent a valuable target for further research and drug development, particularly in the treatment of stress-related mental health disorders. Beaulieu et al. demonstrated that D3R antagonism in the VTA reduces contextual fear memory, whereas D2/D3 agonists alleviate PTSD symptoms [20]. Antagonists that specifically target the D3 receptor, such as YQA14, show promising therapeutic potential due to their high affinity, selectivity, longer half-life, enhanced bioavailability, and metabolic stability [21]. However, the extent to which D3R is involved in fear generalization and its underlying mechanisms remains inadequately understood. Thus, further investigation into the regulatory functions of the D3 receptor in fear memory and anxiety generalization is essential in advancing our knowledge in this area and developing effective interventions for conditions such as PTSD.

## 2. Results

### 2.1. Validation of the Fear Generalization Model

Fear pattern separation refers to the neural capacity to distinguish between similar yet distinct stimuli, enabling differential fear responses. This prevents excessive fear reactions to harmless stimuli, enhancing adaptability and survival. However, intense negative stressors induce a persistent fear state, leading to fear sensitization and generalization, manifesting as inappropriate fear responses in neutral environments due to the individual’s inability to differentiate between closely related stimuli and safe environments. It also leads to an impaired ability to differentiate between threatening and non-threatening environments related to memory schema dissociation deficits, i.e., fear sensitization and generalization, which results in excessive psychological and physiological responses affecting the patient’s ability to live a normal life. This study explored the neural mechanisms underlying fear generalization in PTSD by developing a fear memory generalization model.

**Figure 1:**
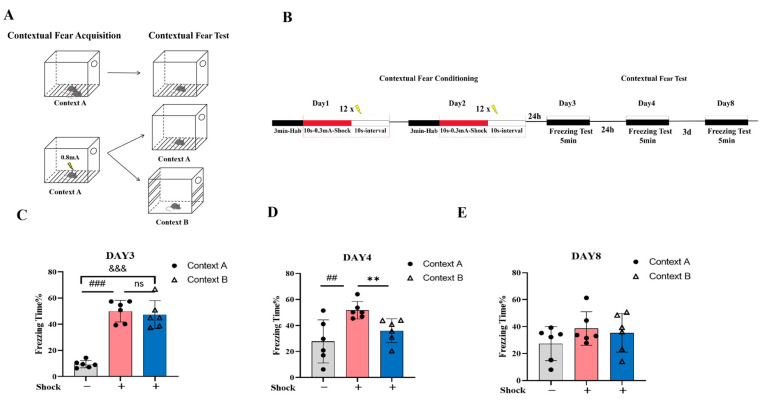
The impact of environmental changes on pattern separation in animals exposed to low-intensity foot-shock. (**A**) The environment for contextual fear conditioning training or testing (Context A) and fear generalization testing in a novel chamber (Context B). (**B**) Experimental design: on day 1 and day 2, mice underwent contextual fear conditioning in the training content, Context A, which consisted of 3 min habituation followed by 12 foot-shocks (10 s, 0.3 mA), each separated by 10 s; on days 3, 4, and 8, mice were placed either back in the training context, Context A, or in a distinct novel context, Context B. (**C**) Contextual fear test on day 3. (**D**) Contextual fear test on day 4. (**E**) Contextual fear test on day 8. One-way ANOVA, Bonferroni test, shock/Context A vs. no-shock/Context A, ^##^ *p* < 0.01, ^###^ *p* < 0.001; shock/Context B vs. shock/Context A, ** *p* < 0.01, shock/Context B vs. no-shock/Context A, ^&&&^ *p* < 0.001. ns, no significant; n = 6 for each group; data are represented as mean ± SEM.

At first, we established a generalized test environment (Figure 1A) to confirm that the modified context was distinct and capable of facilitating pattern separation. In this study, a low-intensity shock (0.3 mA) was initially employed to determine whether the animals could differentiate between the aversive condition and the novel safe context. After two consecutive days of shock administration in Context A, fear generalization assessments were conducted on days 3, 4, and 8 post-conditioning (Figure 1B). The mice receiving aversive foot-shock conditioning in Context A were divided into two groups. During subsequent generalization testing, one group was re-exposed to the original conditioning context (shock/Context A), and another was introduced to a novel, neutral context (shock/Context B). This paradigm allowed for the comparative assessment of fear responses across distinct environments to evaluate potential generalization effects. To control for potential unconditioned fear responses to Context A, we included a naive control group (no-shock/Context A) that underwent identical environmental exposure without foot-shock administration.

On the third day of the acute stress fear test, no significant difference in freezing time was observed between the shock/Context A group and shock/Context B group (Figure 1C, *p* = 0.644), suggesting that low-intensity shocks triggered context-independent freezing, potentially serving as an adaptive protective strategy during acute unpredictable threats. By day 4, the shock/Context B group had exhibited significantly decreased freezing compared to the shock/Context A group (Figure 1D, ** *p* < 0.01), indicating context-specific pattern separation. However, by day 8, no significant differences had been observed between the no-shock/Context A and shock/Context A groups or between the shock/Context A and shock/Context B groups (Figure 1E, shock/Context A vs. shock/Context B, *p* = 0.679), consistent with contextual threat discrimination and the fear extinction-driven suppression of generalized responses. These results suggest that the modified contextual cue can facilitate pattern separation, which may be relevant in studying fear memory generalization.

**Figure 2:**
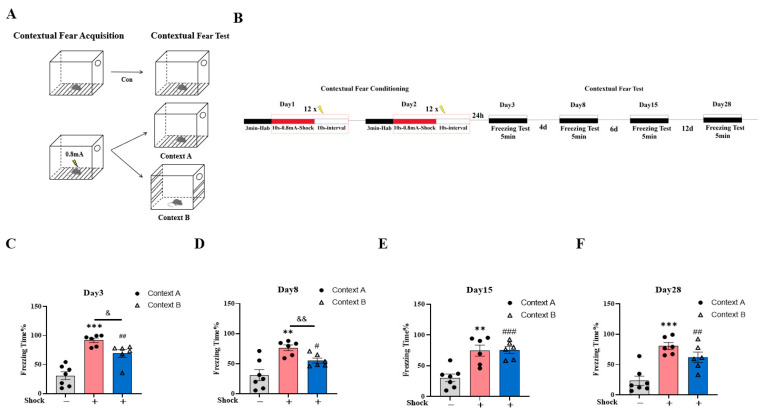
Establishment of generalized animal model of abnormal fear memory. (**A**) The environment for contextual fear conditioning training or testing (Context A) and fear generalization testing in a novel chamber (Context B). (**B**) Experimental design: on day 1 and day 2, mice underwent contextual fear conditioning in the training context, Context A, which consisted of 3 min habituation followed by 12 foot-shocks (10 s, 0.8 mA), each separated by 10 s; on day 3, 8, 15, and 28, mice were placed either back in the training context, Context A, or in a distinct novel context, Context B. (**C**–**F**) Fear response in mice in the conditioning chamber (Context A) and novel chamber (Context B) on day 3 (**C**), 8 (**D**), 15 (**E**), and 28 (**F**). One-way ANOVA, Bonferroni test, shock/Context A vs. no-shock/Context A, ** *p* < 0.01, *** *p* < 0.001; shock/Context B vs. no-shock/Context A, ^#^ *p* < 0.05 ^##^ *p* < 0.01, ^###^ *p* < 0.001, shock/Context B vs. shock/Context A, ^&^ *p* < 0.05, ^&&^ *p* < 0.01. n = 7 for no-shock/Context A group, n = 6 for shock/Context A group and shock/Context B group; data are represented as mean ± SEM.

To assess fear memory generalization under high-intensity aversive conditioning, a 0.8 mA shock stimulus was administered. Animals were assigned to the three groups described previously (shock/Context A, shock/Context B, and no-shock/Context A) using the standardized fear conditioning noted in the method details, with the shock/Context A and shock/Context B groups receiving foot-shocks in Context A on day 1 and 2 and then being exposed to Context A or Context B on day 3, 8, 15, and 28, while the no-shock/Context A group underwent identical exposure procedure to Context A without aversive stimulation on day 1 and 2 (Figure 2A,B).

During generalization testing (day 3, 8, 15, and 28), both the shock/Context A and shock/Context B groups exhibited higher fear responses than the no-shock/Context A group (no-shock/Context A vs. shock/Context A, ** *p* < 0.01, *** *p* < 0.001; no-shock/Context A vs. shock/Context B, ^#^ *p* < 0.05 ^##^ *p* < 0.01, ^###^ *p* < 0.001). However, attenuated fear generalization in shock/Context B (vs. shock/Context A) was restricted to days 3 and 8 (Figure 2C,D, day 3, ^&^ *p* < 0.05; day 8, ^&&^ *p* < 0.01), with no intergroup differences being observed by days 15 and 28 (Figure 2E,F, *p* > 0.05), suggesting that some degree of pattern separation occurred during the early stages of stress.

These findings demonstrate that high-intensity electric shock can significantly induce long-term fear behavior in mice and exhibit long-term fear memory generalization characteristics. This suggests that this model may be valuable in studying the neural circuitry underlying abnormal fear memory.

### 2.2. D3R Antagonism in VTA Attenuates Fear Generalization

**Figure 3:**
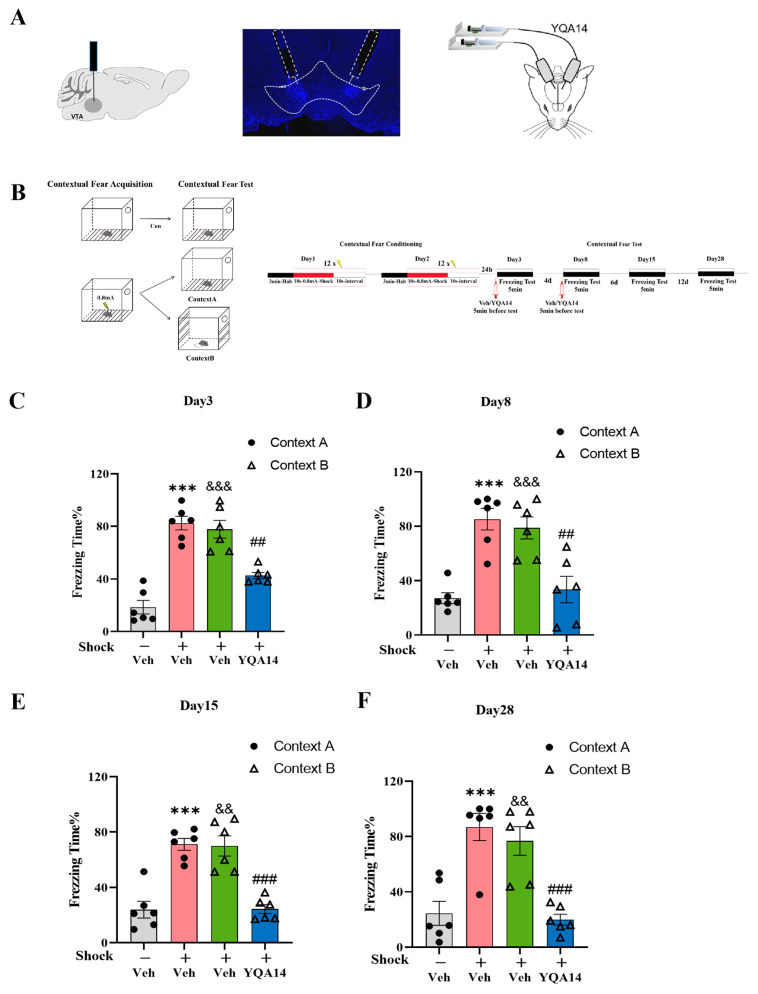
The impact of YQA14 blocking the D3R on fear memory generalization in mice. (**A**) Schematic of cannula implantation in the VTA (left), drug microinjection (right), and representative image of the cannula tip in the VTA (middle). (**B**) Experimental design: on day 1 and day 2, mice underwent contextual fear conditioning in the training chamber (Context A), which consisted of 3 min habituation followed by 12 foot-shocks (10 s, 0.8 mA), each separated by 10 s; on days 3, 8, 15, and 28, mice were placed either back in Context A or in a distinct novel chamber (Context B). Mice were administered Veh or YQA14 before the test on day 3 and day 8. (**C**–**F**) Fear response in mice in the conditioning chamber (Context A) and novel chamber (Context B) on days 3 (**C**), 8 (**D**), 15 (**E**), and 28 (**F**). One-way ANOVA, Bonferroni test, Veh-Shock/Context A vs. Veh-no-shock/Context A, *** *p* < 0.001; Veh-Shock/Context B vs. Veh-no-shock/Context A, ^&&^ *p* < 0.01, ^&&&^ *p* < 0.001, YQA14-shock/Context B vs. Veh-shock/Context B, ^##^ *p* < 0.01, ^###^ *p* < 0.001. n = 6 for each group; data are represented as mean ± SEM.

To investigate whether D3R in the VTA affects the generalization of fear in animals, mice were randomly divided into four groups: (1) Veh-no-shock/Context A (unshocked controls) and (2) Veh-shock/Context A, both receiving bilateral VTA microinfusions of the vehicle (2% DMSO, 0.2 μL per side); (3) Veh-shock/Context B (shocked in Context A, tested in Context B with vehicle); and (4) YQA14-shock/Context B (shocked in Context A, tested in Context B with the selective D3R antagonist YQA14, 0.2 μL per side). All drugs were administered 5 min before fear generalization testing on day 3 and day 8. Figure 3A shows the position of the microinjection cannula in the VTA, while Figure 3B illustrates the experimental design and timeline of this experimental procedure.

The results demonstrate that foot-shock stimuli significantly induced freezing behavior in both the Veh-shock/Context A and Veh-shock/Context B groups compared to the Veh-no-shock/Context A group on days 3, 8, 15, and 28 (*p* < 0.05, *p* < 0.001, Figure 3C–F). The former group demonstrated precise, long-term contextual fear association with Context A, whereas the latter group exhibited persistent fear generalization, reflecting a failure to discriminate between threat-paired and neutral contexts. Critically, YQA14-shock/Context B mice exhibited the sustained attenuation of fear generalization, with significantly reduced freezing behavior compared to Veh-shock/Context B across all test days (*p* < 0.05, *p* < 0.001, Figure 3C–F). The findings indicate that blocking D3R in the VTA during the fear generalization formation period disrupts both acute and long-term fear generalization, highlighting VTA D3R signaling as a critical mediator of maladaptive fear generalization.

### 2.3. D3R Antagonism in VTA Normalizes BLA Hyperactivity in the Fear Generalization Model

**Figure 4:**
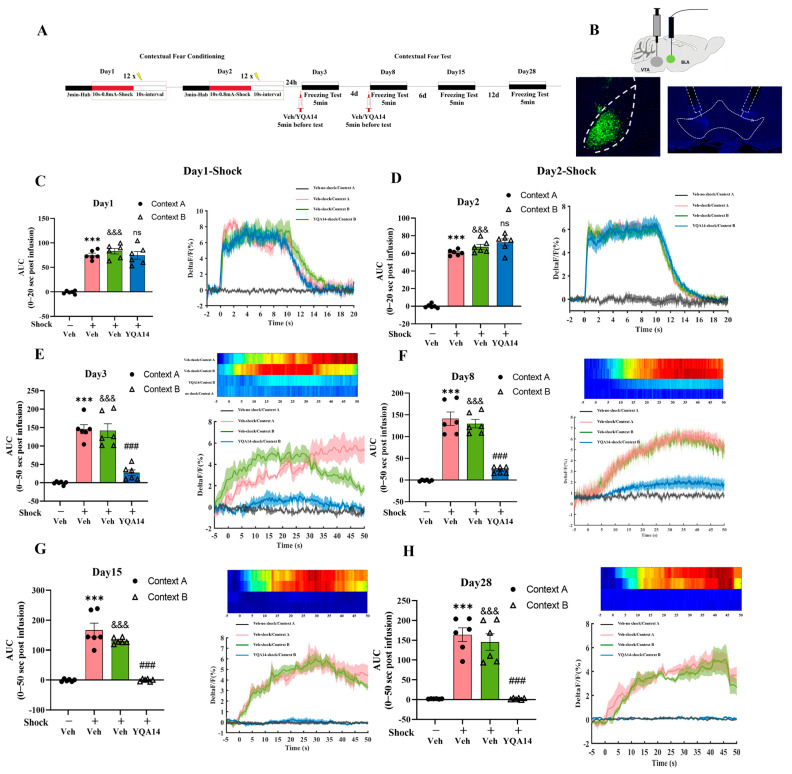
Effect of blocking D3R in the VTA during the latency period after electric shock on BLA neurons and the formation of fear generalization. (**A**) Experimental design: on day 1 and day 2, mice underwent contextual fear conditioning in the training chamber (Context A), which consisted of 3 min habituation followed by 12 foot-shocks (10 s, 0.8 mA), each separated by 10 s; on days 3, 8, 15, and 28, mice were placed back in Context A or in a distinct novel chamber (Context B). Mice were administered with Veh or YQA14 before the test on day 3 and day 8. (**B**) Illustration of viral construct for GCaMP expression in the BLA and cannula implantation in the VTA. (**C**,**D**) The abnormal activation of BLA neurons during the foot-shock period. Left, the AUC during the shock period for each group; right, the average deltaF/F of calcium signals from BLA neurons aligned with foot-shock onset. Solid lines indicate the mean, and shaded areas indicate the SEM. (**E**–**H**) The activity of BLA neurons during fear generalization. Left, the AUC during the contextual fear test for each group; top right, heatmap of deltaF/F ratio of calcium signals from BLA neurons during the first 50 s of the contextual fear test (grouping, from top to bottom: Veh-shock/Context A, Veh-shock/Context B, YQA14-shock/Context B, Veh-no-shock/Context A). Bottom right, the average deltaF/F of calcium signals from BLA neurons during the first 50 s of the contextual fear test. One-way ANOVA, Bonferroni test, Veh-shock/Context A vs. Veh-no-shock/Context A, *** *p* < 0.001; Veh-shock/Context B vs. Veh-no-shock/Context A, ^&&&^ *p* < 0.001, YQA14-shock/Context B vs. Veh-shock/Context B, ^###^ *p* < 0.001. n = 6 for each group; data are represented as mean ± SEM.

To investigate the neural mechanisms underlying D3R-mediated fear generalization, we combined the pharmacogenetic manipulation of the D3R in the VTA with in vivo calcium imaging in the BLA. Figure 4A shows the timeline for conditioning training, fear generalization, and drug administration. GCaMP6m, a genetically encoded calcium indicator, was bilaterally injected into the BLA to monitor neuronal activity, while dual cannulae were implanted in the VTA for drug delivery (Figure 4B). After a two-week viral expression period, 0.2 μL D3R antagonist (YQA14) or vehicle (2% DMSO) per side was microinfused into the VTA prior to fear generalization testing. Consistent with prior groupings (see Section 2.2), fiber photometry recordings were performed during both the conditioning (Context A) and generalization testing (Context A or B) phases to quantify real-time BLA neuronal dynamics.

In conditioning, the fluorescence signal in the BLA of mice receiving foot-shock increased rapidly in response to foot-shock, confirming aversive stimulus-driven hyperexcitability (*p* < 0.001, Figure 4C,D). In subsequent tests, both the Veh-shock/Context A and Veh-shock/Context B groups exhibited sustained BLA hyperactivation across all timepoints (days 3, 8, 15, and 28), correlating with persistent freezing behavior (*p* < 0.001, Figure 4E–H). The acute administration of YQA14 in the VTA in the shock/Context B group significantly attenuated BLA excitability during early generalization tests (days 3 and 8; *p* < 0.001, Figure 4E,F), paralleling reduced freezing. Strikingly, by days 15 and 28, YQA14-treated mice showed complete normalization of BLA activity (Figure 4G,H), reflecting the resolution of fear generalization. These data demonstrate that VTA D3R signaling critically sustains BLA hyperexcitability to drive maladaptive fear memory generalization. Pharmacological D3R inhibition disrupts this circuit-level pathology, normalizing both neuronal dynamics and behavioral fear responses. Blocking D3R offers a therapeutic approach for abnormal fear formation and generalization, providing a potential molecular target for psychiatric disorders associated with fear abnormalities, such as PTSD.

## 3. Discussion

Our previous research suggested a contribution of D3R in contextual fear memory through the blockade of D3R attenuating freezing behavior [22]. Fear generalization is a hallmark of anxiety-related disorders, including PTSD. In this study, we elucidated that the blockade of D3R in the VTA influenced the excitability of neurons in the BLA, which mediated the process of fear generalization. Thus, we speculate that D3R modulates the activity of DAergic neurons in the VTA, influencing the neuronal hyperexcitability of BLA and contributing to fear generalization. This mechanism may explain the therapeutic potential of DRD3 antagonism for use in treating anxiety disorders.

### 3.1. Attenuation of Fear Generalization by Blockade of D3R in VTA

D3R, as an auto-receptor, regulates the excitability of DAergic neurons via negative feedback mechanisms in the VTA [20]. Critically, D3R may dynamically shape DA levels in mesolimbic circuits, thereby influencing adaptive responses to both rewarding and threatening stimuli [23]. In addition, our previous study showed that the DA signal increased after the pharmacological blockade of D3R in the VTA, showing the disinhibition effects of DA neurons in the VTA. Emerging studies have indicated that improvements in resilience to stress attenuate the negative memory formation associated with fear learning [24]. Moreover, the enhancement of homeostasis in the mesolimbic dopamine system can strengthen functional connectivity within the brain’s reward circuitry to ameliorate fear [25]. D3R may be involved in balancing approach–avoidance conflict, underscoring its broader neuro-modulatory role in emotional learning.

### 3.2. D3R Blockade Normalizes BLA Neuronal Hyperexcitability During Fear Generalization

Concurrently, the BLA serves as a central hub for fear processing, integrating sensory and contextual inputs to coordinate fear expression and generalization [26,27,28]. The present results suggest that the blockade of D3R in VTA significantly reduced the neuronal hyperexcitability of BLA during fear generalization tests in the new context. This result aligns with our previous research, which showed a reduction in the increase in DA in the BLA during foot-shock and the retrieval of fear memories with the microinjection of YQA14 into VTA [22]. DAergic signaling within the BLA, mediated primarily through D1 and D2 receptors, is essential to the synaptic plasticity underlying fear memory formation. The dysregulation of DA-BLA communication has been implicated in pathological fear generalization, with the hyperexcitability of BLA projection neurons driving maladaptive fear responses to non-threatening cues [29,30].

The BLA receives dense DAergic projections from the VTA, and DA release in this region potentiates glutamatergic transmission via D1 receptor activation, enhancing neuronal excitability [31,32]. During fear generalization, excessive DAergic signaling in the BLA may drive aberrant synaptic strengthening, blurring the distinction between threat-related and neutral cues [33] by suppressing DA levels in the BLA, thereby mitigating the D1 receptor-mediated excitation of pyramidal neurons. This is consistent with observations that systemic D1 receptor antagonism reduces fear generalization [34]. Additionally, D3R modulation may indirectly influence BLA activity via mesocortical DA pathways. For instance, VTA D3R blockade could enhance prefrontal cortical (PFC) input to the BLA, restoring top-down inhibitory control over hyperactivity [33]. This is critical, as PFC-BLA disconnection is a known contributor to fear generalization [35].

### 3.3. Emotional Regulatory Role of VTA-to-BLA Dopaminergic Projections

DAergic neurons in the VTA regulate the activity of BLA neurons through the D1 and D2 receptor families. Meanwhile, studies on the D3R have shown that blocking D3R enhances the firing of VTA dopaminergic neurons, thereby increasing dopamine release in the BLA. The mechanism behind this phenomenon may be related to the role of D3R in the self-inhibition of dopamine neurons: D3R activity inhibits dopamine release, meaning that blocking D3R enhances release [22]. VTA DAergic neurons exhibit distinct firing patterns in response to rewards and stress, which significantly affect the excitability of BLA neurons. In response to sexual rewards, VTA DAergic neurons increase their firing, and this enhanced firing suppresses GABAergic neurons in the BLA, thereby enhancing the responsiveness of VTA DAergic neurons to reward stimuli [36]. This suggests that VTA dopaminergic neurons form a positive feedback loop by regulating BLA neural activity, enabling individuals to better cope with environmental changes under stress. This mechanism is particularly important in the formation of anxiety and fear memories, as it may influence dynamic changes in dopamine levels in the BLA, thereby modulating an individual’s response to fear stimuli. Our study also found that intervening in VTA-D3R signaling effectively reduces fear generalization, suggesting that D3R has potential therapeutic value in negative emotion regulation.

Additionally, the synaptic transmission properties of VTA-to-BLA dopaminergic projections involve the responses of different neuron types to dopaminergic signals. Studies have shown that neuronal populations in the BLA responding to fear and extinction receive distinct inputs from VTA dopaminergic neurons, which are regulated by different dopamine receptors, thereby influencing the activity patterns of BLA neurons [37]. Enhanced VTA DA signaling promotes the extinction of fear memories, indicating that VTA-to-BLA DAergic projections play a key role not only in learning and memory but also in regulating emotional states.

In summary, the synaptic transmission properties of VTA-to-BLA dopaminergic projections are crucial in understanding emotional regulatory mechanisms, the formation and extinction of fear memories, and generalization processes. Future research focusing on the regulation of D1, D2, and D3 receptors, as well as the dynamics of dopamine release, could further reveal the role of dopamine in mental health disorders and emotional dysregulations, providing new ideas and targets for therapeutic interventions.

### 3.4. Therapeutic Implications and Unresolved Mechanisms

Our findings position D3R as a promising target for interventions aimed at curbing fear generalization in disorders such as PTSD and generalized anxiety. By normalizing BLA hyperexcitability, D3R antagonists could disrupt the neural substrates of maladaptive fear generalization. However, the precise molecular pathways linking VTA D3R activity to BLA plasticity remain elusive. Potential mechanisms include the D3R-mediated regulation of presynaptic DA release probability in VTA-BLA terminals or postsynaptic modulation via D3R heteromerization with other receptors (e.g., D1–D3 complexes) in BLA neurons [38,39,40]. D3R may also interact with stress-related systems, such as corticotropin-releasing factor (CRF) or brain-derived neurotrophic factor (BDNF), to gate BLA excitability [41,42,43]. For example, CRF-DA crosstalk in the VTA-BLA circuit could amplify fear generalization under chronic stress [44], a pathway potentially modulated by D3R. Furthermore, cell-type-specific D3R functions—whether in DA neurons, GABAergic interneurons, or glia—may differentially contribute to behavioral outcomes [45,46,47,48,49]. Future studies employing conditional D3R knockout models and in vivo calcium imaging during fear discrimination tasks are needed to dissect these dynamics [50,51]. While our findings highlight the translational potential of D3R-focused therapies, comprehensive mechanistic insights are imperative in order to optimize specificity and minimize off-target effects.

### 3.5. Strengths and Limitations of the Present Study

This study demonstrates that blockade of D3R in the VTA effectively attenuates the generalization of fear memory. Furthermore, neuronal recordings in the BLA during fear memory retrieval revealed that D3R blockade reverses altered BLA activity patterns. While BLA serves as the downstream brain region for dopamine neuron projections from VTA, we speculate that the dopaminergic circuit of VTA-BLA may be involved in regulating fear generalization. This conclusion is strongly supported by several recent studies. Ding et al. reported that pharmacological inhibition of D3R reduces contextual fear memory and decreases BLA activation, indicating a mechanistic link between VTA dopaminergic signaling and BLA-dependent fear generalization, which is highly consistent with our findings [22]. Chen, M. et al. emphasized the rapid behavioral modulation by neuromodulators in specific brain regions. Their work demonstrated that dopaminergic regulation within limbic and reward-related circuits can rapidly influence emotional memory processing, further supporting our conclusion that targeted blockade of D3R can specifically affect fear generalization without broadly disrupting emotional processing [52]. Our findings strongly suggest functional connectivity between the VTA and BLA, implicating VTA dopaminergic signaling in suppressing excessive fear generalization via modulation of BLA activity.

However, a core limitation of this study is the lack of direct evidence establishing a specific causal role for the VTA-BLA dopaminergic projection in this process. Due to the local diffusion of the drug within the VTA, the observed effects (BLA activity changes and behavioral improvement) may result from altered global VTA activity (affecting all its projection targets) or be mediated through indirect pathways (e.g., affecting other brain regions that subsequently influence the BLA), rather than necessarily reflecting direct actions on dopamine release from VTA-BLA axon terminals onto BLA neurons. Moreover, the somatic expression of D3R in the VTA suggests that the drug action likely occurs by modulating the excitability/output strength of VTA dopaminergic neurons themselves, broadly influencing multiple downstream targets. Concurrently, the study did not identify the specific BLA cell types or receptor mechanisms underlying the observed changes. Thus, while the results strongly implicate the VTA-BLA dopaminergic pathway in regulating fear generalization, they do not definitively establish a direct and necessary causal role for this specific circuit. Therefore, future studies should incorporate more precise circuit manipulation approaches and expand analyses to a broader network level, for instance: (1) Employing retrograde tracing combined with chemogenetic or optogenetic techniques to specifically inhibit or activate VTA dopaminergic neurons projecting to the BLA, assessing whether this recapitulates or blocks the drug effects to directly test the necessity and sufficiency of this projection pathway; and (2) Identifying the specific cell types within the BLA that respond to VTA input. These in-depth investigations are imperative to definitively establish the precise regulatory role of the VTA-BLA dopaminergic circuit in fear generalization and to provide the foundational knowledge for targeted therapies for related psychiatric disorders.

## 4. Materials and Methods

### 4.1. Animals

The experimental subjects consisted of 8~10-week-old adult male C57BL/6J mice, obtained from GemPharmatech Co., Ltd. (Beijing Biotechnology, Beijing, China), under license number SCXK (Beijing) 2023-0008. The mice were housed in specific pathogen-free (SPF) conditions at the animal facility, with the ambient temperature maintained at 22–24 °C and the relative humidity maintained at approximately 50%. A 12-h light/dark cycle was established, with the light phase running from 7:00 to 19:00. Food and water were provided ad libitum, except during experimental procedures. The bedding was replaced two to three times per week to ensure a clean environment. All animal handling and experimental protocols were performed in strict compliance with the Institutional Animal Care and Use Committee (IACUC) guidelines (approval number: IACUC-DWZX-2023-P669).

### 4.2. Chemicals and Administration

Xylazine was obtained from Shanghai Macklin Biochemical Co., Ltd., and ketamine was supplied by the Ministry of Public Security. YQA14 (purity ≥ 99%, white powder) was synthesized at the Beijing Institute of Pharmacology and Toxicology and dissolved in a vehicle solution containing 2% dimethyl sulfoxide (DMSO, assay ≥ 99.7%, Sigma-Aldrich, St. Louis, MO, USA) to achieve a final concentration of 5 mg/mL. YQA14 was bilaterally microinjected into the VTA (0.2 μL per side), with the selected dose determined based on previous studies [22,53,54].

The injection setup comprised a 10 μL Hamilton syringe connected to an injection needle via a polyethylene tube (0.85 mm × 0.42 mm; RWD Life Science, Shenzhen, China). An injection needle extending 0.5 mm beyond the guide cannula was used for precise delivery. The experimental group received YQA14 (0.2 μL per side), while the control group was treated with an equivalent volume of vehicle solution (2% DMSO). An infusion pump (RWD Life Science, Shenzhen, China) delivered the solutions into the VTA at a constant rate of 0.5 μL/min. We monitored the microinjection process by observing the interface between liquid paraffin and the solution within the polyethylene catheter. To ensure the optimal diffusion of the solution from the needle tip, a 1 min dwell time followed each infusion.

### 4.3. The Cannulae and Optic Fiber Implantation Surgeries

Mice were purchased and acclimatized for one week before undergoing brain stereotactic catheterization. The mice were weighed and anesthetized with ketamine (100 mg/kg) and xylazine (12.5 mg/kg) until the absence of the righting reflex and response to toe pinch were confirmed. The surgical procedure was then initiated. The fur on the head, from the eyes to behind the ears, was shaved, and the skin was cleaned and disinfected using iodine solution. A midline incision was made to expose the skull. The mice were positioned on a stereotaxic frame (RWD Life Science, Shenzhen, China), with their incisors secured in the adapter bar and ear bars adjusted to ensure proper alignment. Erythromycin ophthalmic ointment was applied to the eyes to prevent light-induced irritation. The exposed skull surface was cleaned and dried with saline-soaked cotton swabs. The ear bars and nose clip heights were adjusted to ensure that the skull was level along the anterior–posterior axis, with the error not exceeding 0.05 mm.

The stereotaxic coordinates were determined based on the Franklin and Paxinos mouse brain atlas. After the target coordinates were identified, small holes were drilled in the skull, and the dura mater was carefully punctured with a needle. If bleeding occurred during drilling, it was controlled using saline-soaked cotton swabs until no further bleeding was observed. The target coordinates for the BLA and VTA were accessed as follows: VTA (AP: −3.2 mm, ML: ±1.72 mm, DV: −4.1mm, angle: 15°); BLA (AP: −1.4 mm, ML: −4.42 mm, DV: −3.58 mm, angle: 15°). AAV2/9-hysn-GCaMP6m (200 nL) (titer: 5.23 × 10^12^ v g./mL, BrainVTA, Wuhan, China) was injected into the BLA at a rate of 30 nL/min using a microsyringe pump (WPI, Worcester, MA, USA). The injection needle tip was calibrated to zero at the bregma point before being moved to the specified coordinates. After the injection, the needle remained in place for 10 min to ensure the proper diffusion and absorption of the viral vector, and the fiber was buried in the same position. Following the viral injection, a drug delivery catheter was positioned in the VTA. The catheter tip was calibrated at the bregma point, moved to the target site, and fixed in place using cyanoacrylate adhesive (1454 Cyanoacrylates, Tianshan New Material Technology, Beijing, China). Dental cement was used to secure the catheter and fix the fiber optics in place. After the cement had solidified, the apparatus was removed, and the mice were placed on a heated blanket for recovery. Once fully awake, the mice were transferred to their home cages.

### 4.4. Behavioral Assays

#### 4.4.1. Fear Generalization in Contextual Fear Conditioning with Electric Foot-Shock Models in Mice

The shock chamber (dimensions: 30 cm × 23.5 cm × 25 cm, Med Associates Inc., Fairfax, VT, USA) is referred to as Context A. It comprises a transparent polycarbonate roof and anterior surfaces for visual monitoring, sound-attenuating steel side panels, an opaque polymer rear wall, and an electrifiable stainless steel grid floor for precise aversive stimulus administration. During environmental adaptation from Context A (paired with aversive stimuli) to Context B (the unpaired context), the flooring substrate matching the aversive condition was replaced with a rough white plastic board. Lateral steel walls were demarcated with 20 cm wide white adhesive strips to establish a distinct environment.

The detailed protocol is as follows: During the model induction phase (Day 1), the mice were transported to the experimental room and acclimated for 30 min before undergoing the experimental procedures. Following habituation, each mouse was exposed to Context A for 7 min. For shocked cohorts, the session comprised a 3 min acclimation phase followed by 4 min of intermittent shock delivery. Shock delivery commenced at the fourth minute and consisted of 12 cycles alternating between 10 s stimulus application intervals and 10 s stimulus-free intervals. High-intensity shocks were set at 0.8 mA, while low-intensity shocks were delivered at 0.3 mA. Context A was sustained in a dimly illuminated environment to minimize ambient visual interference. The model induction protocol administered on Day 2 mirrored the Day 1 protocol, ensuring consistency across sessions.

Mice were placed in Context A or Context B for 5 min without any electric shocks during the generalization test. A video analysis system was used to track each mouse via behavior recognition and data analysis software (Video Freeze SOF-843, Version 3.1.0.0, Med Associates Inc., USA)) to quantify the duration of freezing behavior and calculate the freezing percentage. Following each conditioning or testing session, the chamber walls and grid floor were cleaned thoroughly with 75% ethanol to eliminate residual olfactory cues from previous subjects, thereby preventing cross-participant sensory contamination.

#### 4.4.2. Microinjection of D3R Antagonist-YQA14 into VTA Before Fear Generalization Test

On the test day, the bilateral cannula drug delivery system was stereotaxically engaged with a guide cannula pre-implanted in the target brain regions in each mouse. Drugs were delivered at a rate of 0.5 μL/min, with a total volume of 0.2 μL per side. The vehicle group was administered with 2% DMSO solution, whereas the YQA14-treated group received 1 μg/0.2 μL/side of YQA14. Previous research by our group [22,55] confirmed that the administration of the identical dosage of YQA14 in the same brain region using the same methodology does not impact spontaneous locomotion in animals and therefore does not interfere with subsequent contextual fear testing. After the completion of the microinjection, the needle was kept in place for 1 min to allow sufficient diffusion of the drug before the mouse was transferred to the test chamber.

#### 4.4.3. Dynamic State in BLA Neurons During Fear Generalization Test

Fiber photometry characterizes neuronal population activity by measuring changes in fluorescence intensity within a specific brain region. The calcium fluorescence signal during the fear generalization test was captured using a dual-color fiber photometry recording system (ThinkerTech, Nanjing, China). The optical fiber cable was pre-bleached for 20 min to minimize interference from its autofluorescence. Following the stabilization of the baseline, the fiber optic cable was attached to the ceramic pins pre-implanted in the BLA; the ceramic optical fiber was cleaned with sterile water beforehand. Fluorescence signals were continuously monitored at 470 nm and 405 nm wavelengths using a sampling frequency of 100 Hz, with an equilibration period of 800 s before the mouse was placed in the shock chamber. The recording continued until the experiment concluded. The fluorescence signal data were processed using MATLAB codes (ThinkerTech, Nanjing, China) and assessed alongside behavioral video recordings to analyze the behavior of the animal.

#### 4.4.4. Fiber Photometry Imaging Data Analysis

The ∆F/F trajectory was generated using a script provided by ThinkerTech Nanjing Biotechnology Co., Ltd. to indicate changes in fluorescence intensity. The calculation was based on the formula ∆F/F0 = (F − F0)/F0, where F represents the current fluorescence intensity and F0 is the baseline fluorescence intensity. For calcium monitoring during fear acquisition (day 1 and day 2), the baseline (F0) was computed as the average signal value in a 2 s period prior to the onset of each shock event. For calcium monitoring during the fear test (days 3, 8, 15, and 28), F0 was derived from the mean signal value over a 5 s temporal window immediately following chamber entry (Context A or Context B). The 405 nm excitation light served as a reference channel during data analysis. MATLAB software (Version R2017b 9.3.0.713579) was used for data preprocessing, including motion correction to remove background noise and the application of a polynomial correction algorithm to compensate for photobleaching effects. Photobleaching refers to the gradual decline in fluorescence signal or autofluorescence in the optical fiber over prolonged recording periods. These adjustments ensured the extraction of accurate fluorescence data. The ∆F/F values are presented as mean values, with the shaded area representing the standard error of the mean (SEM). The analysis results are visualized using heatmaps and line plots.

### 4.5. Statistical Analysis

Statistical analyses were performed using SPSS 26 software (IBM, Armonk, NY, USA), and graphical representations were generated with Prism 8. Data normality and the homogeneity of variance were assessed using the Shapiro–Wilk and Levene tests, respectively. In unequal variances, Welch’s correction was applied to adjust the degrees of freedom. A one-way analysis of variance (ANOVA) was conducted to compare the groups with normally distributed data. When variances were unequal, Welch’s method was applied to adjust the degrees of freedom. Post hoc analyses were carried out using the Bonferroni test for data with homogeneous variances and the Tamhane test for data with heterogeneous variances. All results are expressed as the mean ± standard error of the mean (SEM). Statistical significance was set at *p* < 0.05, with thresholds denoted as follows: * *p* < 0.05, ** *p* < 0.01, and *** *p* < 0.001.

## 5. Conclusions

This study demonstrates that VTA D3R blockade suppresses fear generalization by normalizing pathological hyperexcitability in the BLA, likely through the modulation of DAergic circuits. These findings enhance our understanding of D3R’s dual role in reward and aversion and underscore its therapeutic potential for treating fear-related diseases. However, the molecular and circuit-level intricacies of this regulation demand further exploration. Elucidating how D3R bi-directionally interfaces with neural circuits governing fear resolution versus generalization will pave the way for precision therapies targeting maladaptive fear diseases.

## Data Availability

Data will be made available upon request.
